# Excess of blood eosinophils prior to therapy correlates with worse prognosis in mesothelioma

**DOI:** 10.3389/fimmu.2023.1148798

**Published:** 2023-03-21

**Authors:** Mégane Willems, Arnaud Scherpereel, Eric Wasielewski, Jo Raskin, Hélène Brossel, Alexis Fontaine, Mélanie Grégoire, Louise Halkin, Majeed Jamakhani, Vincent Heinen, Renaud Louis, Bernard Duysinx, Malik Hamaidia, Luc Willems

**Affiliations:** ^1^ Laboratory of Molecular and Cellular Epigenetics (GIGA at University of Liege), Sart-Tilman, Molecular Biology, Teaching and Research Centre (TERRA), Gembloux, Belgium; ^2^ Department of Pneumology and Thoracic Oncology, (CHU Lille) and INSERM (ONCOTHAI), Lille, France; ^3^ Department of Pulmonology and Thoracic Oncology, Antwerp University Hospital, Edegem, Belgium; ^4^ Department of Pneumology, University Hospital of Liege, Liege, Belgium

**Keywords:** malignant pleural mesothelioma, eosinophils, chemotherapy, cisplatin, pemetrexed

## Abstract

**Background:**

Only a fraction of patients with malignant pleural mesothelioma (MPM) will respond to chemo- or immunotherapy. For the majority, the condition will irremediably relapse after 13 to 18 months. In this study, we hypothesized that patients’ outcome could be correlated to their immune cell profile. Focus was given to peripheral blood eosinophils that, paradoxically, can both promote or inhibit tumor growth depending on the cancer type.

**Methods:**

The characteristics of 242 patients with histologically proven MPM were retrospectively collected in three centers. Characteristics included overall survival (OS), progression-free survival (PFS), overall response rate (ORR) and disease control rate (DCR). The mean absolute eosinophil counts (AEC) were determined by averaging AEC data sets of the last month preceding the administration of chemo- or immunotherapy.

**Results:**

An optimal cutoff of 220 eosinophils/µL of blood segregated the cohort into two groups with significantly different median OS after chemotherapy (14 and 29 months above and below the threshold, *p* = 0.0001). The corresponding two-year OS rates were 28% and 55% in the AEC ≥ 220/µL and AEC < 220/µL groups, respectively. Based on shorter median PFS (8 *vs* 17 months, *p* < 0.0001) and reduced DCR (55.9% vs 35.2% at 6 months), the response to standard chemotherapy was significantly affected in the AEC ≥ 220/µL subset. Similar conclusions were also drawn from data sets of patients receiving immune checkpoint-based immunotherapy.

**Conclusion:**

In conclusion, baseline AEC ≥ 220/µL preceding therapy is associated with worse outcome and quicker relapse in MPM.

## Introduction

Malignant pleural mesothelioma (MPM) is a cancer associated with very poor prognosis mainly induced by occupational exposure to asbestos fibers (1). Despite the ban or limitation of asbestos use (2), incidence of MPM is still increasing worldwide (3) due to the long latency time between exposure and neoplasm development. There are 3 main histological subtypes of MPM: epithelioid (60–80% of cases), sarcomatoid (< 10%) and biphasic/mixed (10–15%) (4, 5). Therapeutic standard options include conventional treatments (surgery, radiotherapy, chemotherapy) and, more recently, immunotherapy (6–8). Thus, since 2003 the first-line standard-of-care for unresectable MPM has been chemotherapy based on the combination of a DNA cross-linking agent (cisplatin or carboplatin) and an antifolate (pemetrexed) (6). The median overall survival (mOS) obtained with this regimen ranges between 13 and 16 months (6, 9). Addition of an anti-VEGF antibody (bevacizumab) to cisplatin/pemetrexed improved mOS up to 18.8 months compared to 16.0 months in the control arm (9). As many MPM patients have a weakened immune system, chemotherapy initially seemed to be a better option than immunotherapy (10). However, the recent first-line dual immunotherapy by immune checkpoint inhibitors (ICIs) (nivolumab and ipilimumab, targeting PD-1 and CTLA-4, respectively) extended mOS from 14.1 months with standard chemotherapy to 18.1 months (11). Immunotherapy has only a limited benefit for the epithelioid subtype but is particularly effective for non-epithelioid MPM (12). Compared with chemotherapy, ICIs clearly provide much better OS rates at 4 years in non-epithelioid MPM (i.e., 14% *vs* 1%, respectively).

Despite these recent improvements, the prognosis of MPM remains globally poor. The biological mechanisms that drive the effectiveness of available therapies are still not well understood. However, the recent breakthroughs of ICIs indicate that the tumor microenvironment (TME) is a major parameter in cancer development and response to therapy. Even though mesothelioma was initially considered as a “cold” tumor (*i.e.*, absence of T cells within or at the edges of the tumor), the paradigm has recently been revisited (10). In the mesothelioma TME, tumor-associated macrophages (TAMs) are the most abundant immune infiltrating cells (13–18). The phenotype of these TAMs is shaped by mediators expressed by tumor cells. Therefore, the ability of TAMs to orchestrate the innate immune response and to modulate activation of effector T-cells is impaired in MPM. Among immune cells that regulate macrophage polarization, eosinophils favor the M1 phenotype through the production of IFN-γ and TNF-α. However, eosinophil-derived IL-4 and IL-13 can also promote suppressive TAMs and shape the TME (19, 20). The balance between Th1- and Th2-related cytokines modulates the migration and activation of CD8^+^ T-cells and affects the local anti-tumor response. Among their pleiotropic activities, eosinophils also promote angiogenesis and tissue healing *via* VEGF, FGF and PDGF production. Besides their ability to shape the TME through the expression of cytokines, eosinophils display cytotoxic effects by secreting granule proteins and granzyme A.

Altogether, this evidence thus indicates that eosinophils exert both pro- and anti-tumorigenic activities. The final outcome will depend on a variety of parameters that include the cytokine balance, the interaction of eosinophils with other immune cells and the resulting cytotoxicity against the tumor. In this context, we investigated the correlation of blood eosinophil counts with mOS, progression-free survival (PFS) and duration of response in patients undergoing chemo- or immunotherapy.

## Materials and methods

### Patients’ selection and data collection

Two hundred and forty-two eligible MPM patients were included in this study. Between January 2009 and December 2021, these patients were given chemo- or immunotherapy in 3 hospitals: 68 at the University Hospital of Liege (Belgium), 61 at the University Hospital of Antwerp (Belgium) and 101 at the University Hospital of Lille (France). According to standard guidelines, 230 patients received cisplatin or carboplatin and pemetrexed as first-line chemotherapy (4, 21). Among these, 32 patients also received 2^nd^ or 3^rd^ line immunotherapy with nivolumab and ipilimumab. Twelve patients were given ICIs in first-line therapy.

Exclusion criteria included autoimmune disease, congenital or acquired immunodeficiency including HIV, asthma, and active parasitic infection at diagnosis, requiring systemic treatment. Patients diagnosed less than a year before the study was initiated or who did not complete a full treatment plan were also excluded as the follow-up period was too short.

All data were collected for medical purposes and obtained retrospectively. The following data were collected from hospital databases: date of birth; date of diagnosis; sex; histological subtype; BAP-1 deletion; date and type of treatment; response to treatment at 3 months, 6 months and 1 year; hematological lab tests before, during and after treatment; smoking status; diabetes status; asbestos exposure information; comorbidity information; date of death if applicable. Clinical staging was not available for most patients.

This study was performed in compliance with the Helsinki Declaration and was approved by the local Ethics Committee with the reference 2020/45 (University Hospital of Liege) and 2022/1844 (University Hospital of Antwerp) and declared to the local Data Protection Officer (DPO), per General Data Protection Regulation (University Hospital of Lille). As this was a retrospective and non-interventional study, informed consent was not required. Medical records were analyzed pseudonymously.

### Outcomes and statistical analysis

Absolute eosinophil counts (AEC) are routinely determined from hemograms collected at presentation. They were retrieved from the available medical records. Optimal AEC cutoff was determined with the X-tile 3.6.1 software (Yale University, New Heaven, CT) and validated by the receiver operating characteristics (ROC) curve. The analysis was based on the mean AEC, averaged during the last month preceding the administration of chemo- or immunotherapy.

The primary studied endpoint was mOS, defined as the time from the diagnosis to the date of death due to any cause. Secondary endpoints included PFS, response rate, duration of response and disease control rate. The response was assessed with radiographic tumor assessment according to the modified Response Criteria (mRECIST) [version 1.1] (4, 22). PFS was defined as the time between diagnosis and first-documented tumor progression or death due to any cause, whichever came first. Response rate was defined as the best overall response of complete response (CR) or partial response (PR). Duration of response was defined as the time from the first response to the first documented tumor progression or death due to any cause, whichever occurred first. Disease control rate was defined as the best overall response of CR, PR, or stable disease (SD).

Hazard ratios (HRs) and confidence intervals (CIs) of 95% were assessed using an unstratified Cox proportional hazards regression model. Survival curves and rates were estimated with the Kaplan-Meier method and Log-Rank test. Patients with missing values were excluded from the analysis. For statistical purposes, age was categorized as less than 65 years and more or equal to 65 years, whereas subtype was classified as epithelioid and non-epithelioid (i.e., sarcomatoid, biphasic or desmoplastic).

Statistical analysis and graphs were performed by using Prism GraphPad 8 or RStudio 2022.07.1 + 554.

## Results

### A threshold of AEC at 220/µL splits the cohort into two groups with different overall survival

**Figure 1:**
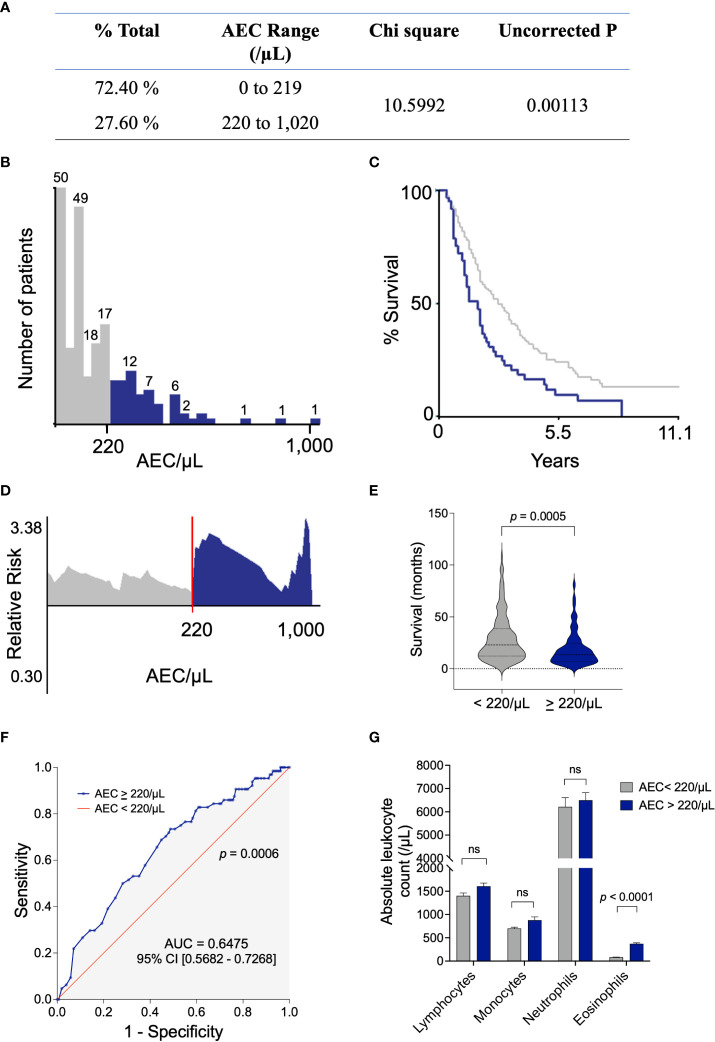
Determination of the AEC cutoff that optimally segregates the cohort according to OS. **(A)** An average AEC was calculated for each patient using the counts of the last month preceding the first administration of chemotherapy. The X-tile 3.6.1 software divided the data set into two populations by selecting significative uncorrected p-value and the highest Chi square. **(B)** Distribution of patients according to their AEC (below 219 per μl of blood in grey and 220-1,020 in blue). **(C)** Kaplan-Meier survival curve according to the AEC < 220/µL and AEC ≥ 220/µL. **(D)** The relative risk estimated by dividing the death incidence at each AEC by the death incidence of the population. **(E)** Median survival (in months) of the populations according to the AEC threshold. Normality of the populations was analyzed by the Shapiro-Wilk test and distributions were compared by Mann-Whitney test. **(F)** The ROC analysis of the true (sensitivity) and false (1-specificity) positive rates. **(G)** Absolute leucocyte counts (mean +/- standard deviation) in patients with AEC < 220/µL and AEC ≥ 220/µL. Statistical significance was calculated with the unpaired t-test. AEC, absolute eosinophil count; ROC, receiver operating characteristics; AUC, area under the curve.

The X-tile software was used to identify the optimal AEC cutoff associated with survival in the cohort of 230 MPM patients receiving first-line chemotherapy. This bioinformatic tool is a graphical method for biomarker assessment and outcome-based cut-point optimization (23). The program provides the optimal division of the data by selecting significant uncorrected p-value and the highest Chi-square. An average AEC was calculated for each patient using the counts of the last month preceding the first administration of chemotherapy. The optimal AEC cutoff determined with the X-tile software was 220 eosinophils/µL of blood (Chi-square = 10.5992, uncorrected *p* = 0.00113, **Figure 1A**). This threshold divided the cohort into two groups of 169 (72.40%) and 61 (27.60%) subjects with AEC < 220/µL (in grey) and AEC ≥ 220/µL (in blue), respectively (**Figure 1B**). These settings optimally segregated the Kaplan-Meier survival curves of the two subsets (**Figure 1C**). The relative risk was estimated by dividing the death incidence corresponding to each AEC by that of the population (**Figure 1D**). The AEC 220/µL cutoff classified the patients into two populations with highly significant different distributions (*p* = 0.0005, **Figure 1E**). The ROC curve illustrating the true (sensitivity) and false (1-specificity) positive rates validated the cutoff of 220 eosinophils/µL of blood (AUC = 0.6475, *p* = 0.0006, **Figure 1F**).

To verify that the measured AEC levels did not result from an increase of all white blood cells, the average absolute counts of other leukocytes were calculated. In populations with AEC < 220/µL and AEC ≥ 220/µL, the absolute counts of lymphocytes, monocytes and neutrophils were similar (**Figure 1G**). Since the absolute counts of eosinophils differed significantly (*p* < 0.0001), it was concluded that high levels of AEC did not result from a general increase of all leukocyte subsets. Furthermore, X-tiles analysis of neutrophils, lymphocytes, monocytes and neutrophil-to-lymphocyte ratio (NLR) did not highlight any threshold or correlation with mOS.

This cut-point selection analysis thus indicated that a threshold of AEC ≥ 220/µL within the last month preceding the first administration of chemotherapy optimally divided the total population into two subsets displaying statistically significant different overall survival times.

### Study population

Among the 230 eligible patients treated by chemotherapy, 53 males and 8 females’ cases were above the threshold of AEC ≥ 220/µL (**Table 1**). The median age at the time of diagnosis of the patients with AEC < 220/µL and AEC ≥ 220/µL was similar (67 +/- 10.4 *vs* 67 +/- 10.9 years, respectively). In both categories, most patients were male (74.0% and 86.9%) and presented an epithelioid subtype of MPM (87.0% and 77.0%). These characteristics were thus representative of typical gender and histologic distributions of MPM (4).

**Table 1:** Baseline characteristics of all patients receiving chemotherapy, segregated by the AEC cutoff of 220/µL.

	AEC < 220/µL		AEC ≥ 220/µL	
	*N* of patients (total 169)	% of patients	*N* of patients (total 61)	% of patients
**Age at diagnosis:**	67 ± 10.4 years		67 ± 10.9 years	
Sex				
Male	125	74.0%	53	86.9%
Female	44	26.0%	8	13.1%
Histological subtype				
Epithelioid	147	87.0%	47	77.0%
Sarcomatoid	10	5.9%	7	11.5%
Biphasic	6	3.6%	5	8.2%
Desmoplastic	3	1.8%	1	1.6%
Unknown	3	1.8%	1	1.6%
Known asbestos exposure				
Yes	48	28.4%	21	34.4%
No	91	53.8%	25	41.0%
Unknown	36	21.3%	15	24.6%
ECOG status prior to chemotherapy				
0	28	16.6%	8	13.1%
1	84	49.7%	26	42.6%
2	6	3.6%	2	3.3%
Unknown	57	33.7%	25	41.0%
Smoking status				
Smoking	25	14.8%	11	18.0%
Detoxed	56	33.1%	14	23.0%
No	78	46.2%	35	52.5%
Unknown	10	5.9%	4	6.6%
Diabetes				
Insulin-dependent	13	7.7%	5	8.2%
Non-insulin-dependent	19	11.2%	3	4.9%
No	128	75.7%	51	83.6%
Unknown	9	5.3%	2	3.3%
BAP-1 loss of expression				
Yes	42	24.9%	11	18.0%
No	17	10.1%	4	6.6%
Unknown	110	65.1%	46	75.4%

Due to limitations of a retrospective study, only partial information was available for asbestos exposure, Eastern Cooperative Oncology Group (ECOG) performance status prior to chemotherapy, smoking status, diabetes, and BAP-1 expression (**Table 1**). Prior exposure to asbestos was confirmed in 28.4% and 34.4% of patients with AEC < and ≥ 220/µL, respectively. The proportions of patients presenting different ECOG performance status were similar. OS and AEC/μL were not statistically different in patients with ECOG status 0, 1 and 2 (**Supplementary Figure 1**). Both active tobacco consumption and diabetes affected a minority of patients. Loss of BAP-1 expression determined by immunohistochemistry was validated in 24.9% of AEC < 220/µL and 18.0% of AEC ≥ 220/µL subsets.

It thus appeared that the two populations split by the AEC 220/µL cutoff shared similar characteristics of age, gender and histological subtype.

### AEC ≥ 220/µL is correlated with shorter overall survival

**Figure 2:**
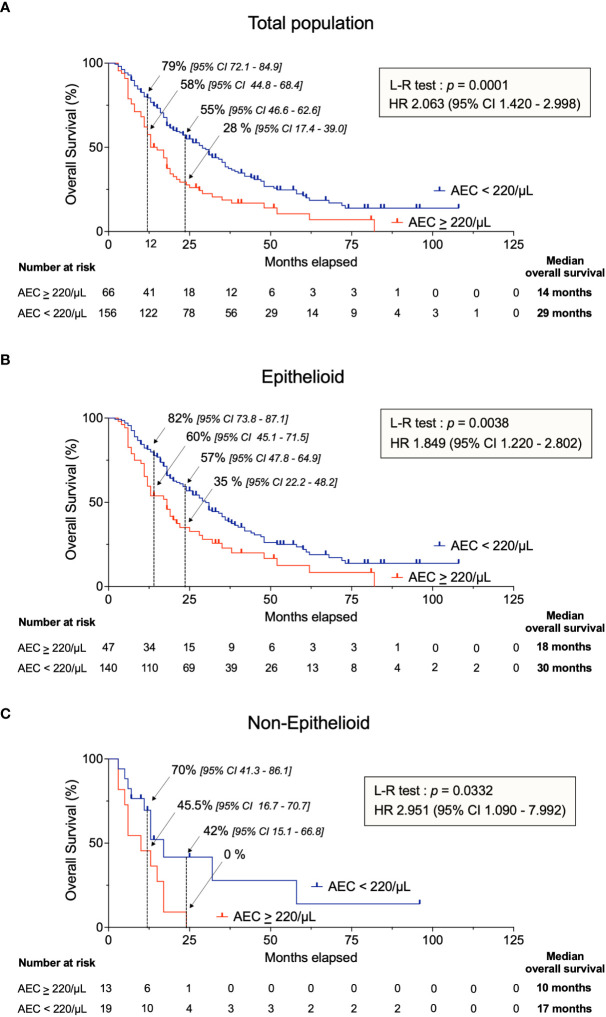
Kaplan-Meier analysis of OS in chemotherapy-treated patients with AEC ≥ 220/µL (in red) and AEC < 220/µL (in blue). **(A)** All patients of the cohort, **(B)** epithelioid MPM and **(C)** non-epithelioid MPM. AEC, absolute eosinophil count; OS, overall survival; L-R test; Log-Rank test; HR, hazard ratio; CI, confidence interval.

Kaplan-Meier analysis showed that patients characterized by AEC ≥ 220/µL during the month preceding their chemotherapy had a highly significant shorter OS compared to subjects with AEC < 220/µL (**Figure 2A**). The mOS of the 230 individuals enrolled in this study were 14 months and 29 months for AEC above or equal to and below 220/µL, respectively (*p* = 0.0001, HR of 2.063 [95% CI 1.420 – 2.998]). At 1 year, the OS rates were 58% [44.8 – 68.4] in subjects with AEC ≥ 220/µL compared to 79% [72.1 – 84.9] in the AEC < 220/µL group. The difference between the two categories was more pronounced at 2 years (28% [17.4 – 39.0] *vs* 55% [46.6 – 62.6]) corresponding to a 2.0-fold improvement in mOS when AEC < 220/µL. The lower mOS in the AEC ≥ 220/µL subset was observed independently of the histologic subtype (**Figures 2B, C**).

Although the proportion of patients with AEC ≥ 220/µL differed in the 3 hospitals (i.e., 17.8% in Lille, 32.35% in Liege and 34.4% in Antwerp; **Supplementary Table 1**), the mOS was significantly reduced from 36 to 17 months (*p* = 0.0062 for CHU of Lille) and from 29 to 16 months (*p* = 0.0184 for UZ Antwerp) (**Supplementary Figure 2**). However, there was no statistical difference in patients from the Liege CHU (17 vs 15 months, *p* = 0.4610) which may indicate a center bias. Furthermore, OS was shorter for patients with AEC ≥ 220/µL in predefined subgroups (**Supplementary Figure 3**).

Altogether, this retrospective observational study thus indicated that MPM patients with AEC ≥ 220/μL had a shorter mOS.

### AEC superior or equal to 220/μL is associated with earlier relapse

**Figure 3:**
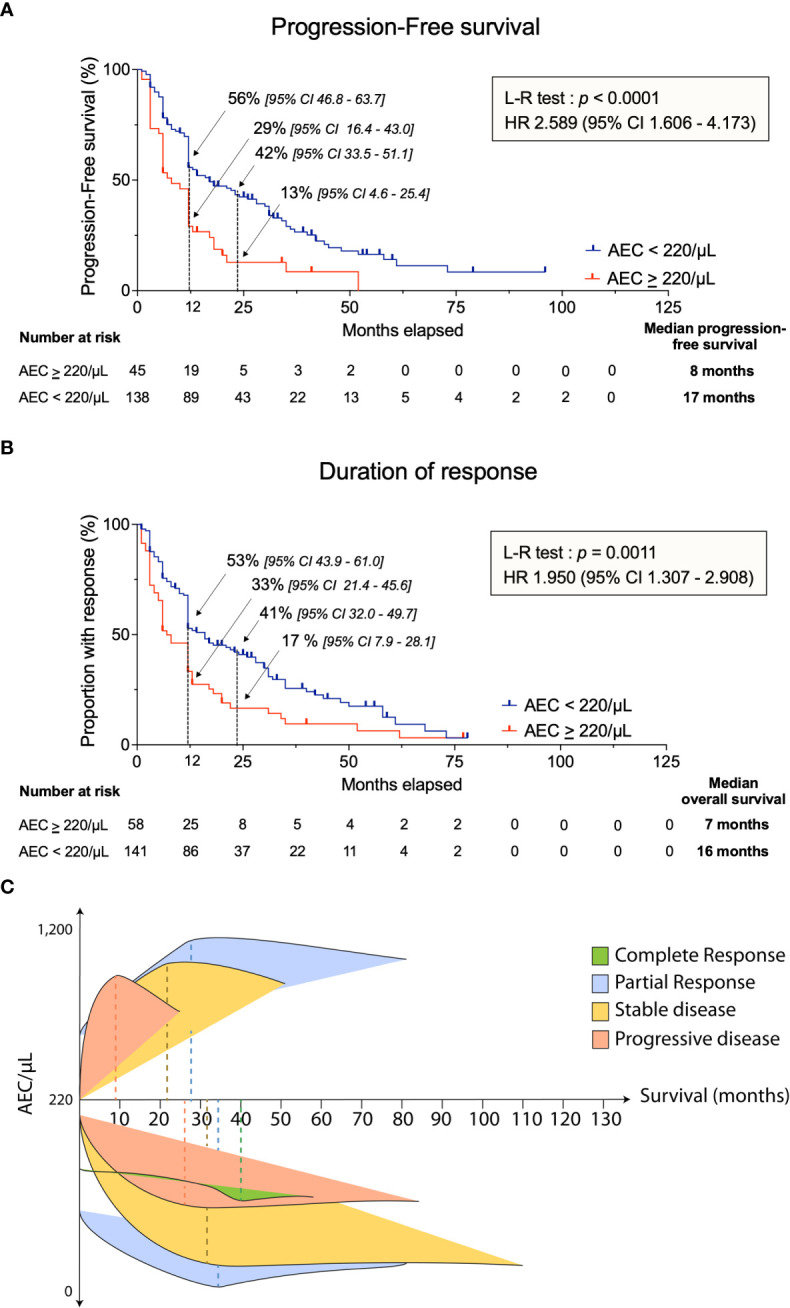
Response to chemotherapy according to AEC cutoff. **(A)** Progression-free survival in all randomized patients. **(B)** Duration of response in confirmed responders segregated by the AEC cutoff of 220/µL. **(C)** Schematic representation of response to chemotherapy, survival distribution and AEC. Dashed lines are the median survival (in months) corresponding to the type of response. L-R test, Log-Rank test; HR, hazard ratio; AEC, absolute eosinophil count.

The median PFS after chemotherapy was significantly lower in the AEC ≥ 220/μL group compared to the AEC < 220/μL subset (8 months *vs* 17 months; *p* < 0.0001, HR 2.589 [1.606 – 4.173]) (**Figure 3A**). Notably, PFS at 2 years was 13% [4.6 – 25.4] *vs* 42% [33.5 – 51.1] in patients with AEC ≥ 220/μL and AEC < 220/μL, respectively. Furthermore, the median time until progression or relapse differed significantly (7 months when AEC ≥ 220/μL *vs* 16 months when AEC < 220/μL; *p* = 0.0011, HR 1.950 [1.307 – 2.908]) (**Figure 3B**). Analysis of this retrospective dataset thus indicated that relapse after chemotherapy occurred more rapidly when AEC ≥ 220/μL.

Partial information on response to treatment was available in the retrospective data set (145 and 45 patients in the AEC < 220/μL and AEC ≥ 220/µL groups, respectively (**Table 2**). Information on response to treatment was missing in 31 patients. A single CR was observed in each category, consistently with other MPM trials (11, 24, 25). The objective response rate (ORR) combining CR and PR was similar in the 2 subsets (15.9% *vs* 20.4% at 3 months and 9.0% *vs* 9.3% at 6 months). In contrast, SD was significantly more common in patients with AEC < 220/μL (56.6%) than in those with AEC ≥ 220/µL (38.9%). This difference in SD was due to a higher proportion of patients with progressive disease (PD) in the AEC ≥ 220/µL subgroup (33.3%, *vs* 17.2%). Only 33% [21.4 – 45.6] of patients with AEC ≥ 220/μL displayed a disease control, including CR, PR and SD of at least 1 year, compared to 53% [43.9 – 61.0] in subjects with AEC < 220/μL. This difference was still observed after 2 years (17% in AEC ≥ 220/µL *vs* 41% in AEC < 220/μL).

**Table 2:** Summary of patient’s response in all randomized patients receiving chemotherapy, segregated by the AEC cutoff of 220/µL.

	AEC < 220/µL		AEC ≥ 220/µL	
	*N* of patients (total 145)	% of patients	*N* of patients (total 54)	% of patients
Best overall response				
Complete response	1	0.7%	1	1.9%
Partial response	37	25.5%	14	25.9%
Stable disease	82	56.6%	21	38.9%
Progressive disease	25	17.2%	18	33.3%
Disease control rate (CR + PR + SD)				
3 months	99	68.3%	29	53.7%
6 months	81	55.9%	19	35.2%
Objective response rate (CR + PR)				
3 months	23	15.9%	11	20.4%
6 months	13	9.0%	5	9.3%
Proportion of patients with a response of at least 1 year				
1 year	53%		33%	
95% CI	43.9 – 61.0		21.4 – 45.6	
2 years	41%		17%	
95% CI	32.0 – 49.7		7.9 – 28.1	

Responses were assessed accordingly to mRECIST v1.1 criteria. CR, complete response; PR, partial response; SD, stable disease; CI, confidence interval.

Together, these data showed that the AEC cutoff of 220/µL identified groups of patients with different mOS (**Figure 2A**) and response to chemotherapy (**Figure 3C**). The same conclusion was drawn when the study was extended to patients who received immunotherapy (**Supplementary Figures 4**, **5**). Indeed, Kaplan-Meier analysis highlighted that, patients with AEC ≥ 220/µL prior to immunotherapy had a shorter OS (*p* = 0.0022) and was characterized by a higher proportion of PD (42.9% *vs* 18.9%) compared with the AEC < 220/µL group.

## Discussion

In this report, we showed that patients with an AEC ≥ 220/µL prior to their therapy appear to have a worse outcome and relapse more rapidly. Importantly, we have considered the mean AEC value measured during the month preceding administration of chemo- or immunotherapy. In particular, the disease control rate was improved in chemotherapy-treated patients with AEC < 220/µL and, consistently, the proportion of subjects with a response at two years was increased by 2.4-fold (i.e., 41% *vs* 17%, **Table 2**). While the proportion of patients with objective response rate (CR + PR) was similar above and below the threshold of AEC 220/µL, there was a statistically significant difference of SD (**Table 2**; **Supplementary Figures 4C and 5C**).

It should be mentioned that, in this study, we excluded patients with hypereosinophilia induced by asthma, allergy, parasitic infection, autoimmune disease, and medication (26, 27). Indeed, these conditions require systemic treatments that would have affected the immune system. It should also be noted that, within the “normal” range (0-450 eosinophils/µL of blood), there is no clear mechanism that explains the fluctuations of eosinophil levels.

In this retrospective study, successive CT evaluations and over time distinguishable tumor margins were often missing. It should however be mentioned that multiple radiographic assessments are particularly challenging in MPM (28). Therefore, OS is preferred and considered to be a more objective and reliable endpoint compared to PFS, response rate and duration of response (11). In this perspective, we showed that the AEC 220/μL threshold predicted a significant difference in mOS (14 *vs* 29 months in patients treated with chemotherapy and 25 *vs* 48 months with immunotherapy, **Figure 2** and **Supplementary Figure 4**). The significant association between AEC and OS does not preclude that eosinophilic MPM patients could still respond to chemotherapy or ICIs (29). Consistently, MPM case reports of poor response and fast deterioration have been described in eosinophilic patients (29–31). If validated by prospective and interventional studies, this conclusion could thus be of particular interest for MPM management.

In fact, the association of AEC and OS has been investigated in other cancers, yielding to opposite conclusions. Indeed, excess of eosinophils in the peripheral blood has been correlated with either a better or a worse prognosis depending on the cancer type (20, 32, 33). For example, in non-small cell lung cancer (NSCLC) and melanoma, an AEC equal or superior to 300/µL measured before therapy was associated with a better outcome (34–43). By contrast, the level of peripheral blood eosinophils is an independent prognostic factor for disease progression and disease-specific death in Hodgkin’s lymphoma and primary cutaneous T-cell lymphoma (40, 44–46).

Due to the more recent advent of immunotherapy in MPM, the number of first-line immunotherapy-treated patients included in this study was limited. However, the difference of OS in the AEC ≥ 220/µL and AEC < 220/µL groups was nevertheless statistically significant (L-R test *p* = 0.0022; **Supplementary Figure 4**). This conclusion was valid providing that AECs were determined before, but not during or after, the initiation of therapy. In contrast, increase of peripheral blood eosinophils during treatment with ICIs is associated with better response and clinical outcome in NSCLC, indicating that the correlation could be dependent on the tumor type (47, 48). Although the biological mechanisms underlying this difference are still not well understood, it is likely that the TME is a central parameter of this cancer specificity. The TME most likely shapes the phenotype of eosinophils into diverse subpopulations with opposite functions, as illustrated in asthma (49–51). In MPM, the interaction of eosinophils with other immune cells such as macrophages, monocytes and neutrophils may direct pro- or anti-tumor functions as well as response to therapy (13–18). Consistently, inflammation markers such as lymphocyte predominance, NLR and absolute monocyte count (AMC) have been correlated with poor survival (52–57). Analysis of the data set of our cohort did not reveal any association of OS with NLR, AMC and monocyte-to-lymphocyte ratios.

Although a causal link still needs to be demonstrated, the correlation between AEC and OS possibly opens direct prospects for therapeutic intervention. Indeed, our report suggests that there might be a benefit to decrease the AEC below the 220/μL threshold before initiating the chemo- or immunotherapy. For example, glucocorticoids (e.g., methylprednisolone) used to prevent pemetrexed-associated rash, emesis and inflammation (58–60) are able to induce apoptosis of eosinophils (61). In our study, a single dose of methylprednisolone at 48mg effectively reduced inflammation but did not reduce myeloid cell counts as numbers remained approximately constant before and after administration. More specific approaches targeting eosinophils have recently been developed in the treatment of asthma (62). Monoclonal antibodies interacting with cytokines associated with eosinophilia (e.g., IL-5, IL-33) are currently evaluated in clinical trials to treat eosinophilic COPD patients: Mepolizumab (anti-IL-5; NCT04075331), MEDI3506 (anti-IL-33; NCT04570657), REGN3500 (anti-IL-33; NCT04701983 and NCT04751487) and Astegolimab (anti-ST2; NCT03615040). Whether these targeted approaches are effective as add-on therapy in MPM could thus merit further evaluation.

## Conclusion

In summary, this retrospective study shows that an AEC threshold of 220/µL measured prior to therapy identifies populations with distinct outcomes in mesothelioma, supporting further prospective analysis and possibly interventional trials.

## Data availability statement

The original contributions presented in the study are included in the article/**Supplementary Material**. Further inquiries can be directed to the corresponding author.

## Ethics statement

The studies involving human participants were reviewed and approved by University Hospital of Liège Ethical Committee, reference 2020/45 University Hospital of Antwerp Ethical Committee, reference 2022/1844. Written informed consent for participation was not required for this study in accordance with the national legislation and the institutional requirements.

## Author contributions

MW, AS and EW collected the dataset in Lille CHU. JR collected the dataset in Antwerp CHU. MW, AF and MH collected the dataset in Liege CHU. AF, AS, EW, HB, JR, MG, LH, MJ, VH, RL participated in data interpretation and manuscript reviewing. MW, LW and MH designed the study. MW and LW drafted the manuscript. All authors contributed to the article and approved the submitted version.

## References

[1] Asciak R George V Rahman NM. Update on biology and management of mesothelioma. Eur Respir Rev (2021) 30:1–13. doi: 10.1183/16000617.0226-2020 PMC9489032 33472960

[2] Kazan-Allen L. Current asbestos bans (2022). Available at: http://www.ibasecretariat.org/alpha_ban_list.php (Accessed September 14, 2022).

[3] Bray F Ferlay J Soerjomataram I Siegel RL Torre LA Jemal A. Global cancer statistics 2018: GLOBOCAN estimates of incidence and mortality worldwide for 36 cancers in 185 countries. CA Cancer J Clin (2018) 68:394–424. doi: 10.3322/caac.21492 30207593

[4] Popat S Baas P Faivre-Finn C Girard N Nicholson AG Nowak AK. Malignant pleural mesothelioma: ESMO clinical practice guidelines for diagnosis, treatment and follow-up. Ann Oncol (2022) 33:129–42. doi: 10.1016/j.annonc.2021.11.005 34861373

[5] Alì G Bruno R Fontanini G. The pathological and molecular diagnosis of malignant pleural mesothelioma: A literature review. J Thorac Dis (2018) 10:S276–84. doi: 10.21037/jtd.2017.10.125 PMC5830567 29507796

[6] Vogelzang NJ Rusthoven JJ Symanowski J Denham C Kaukel E Ruffie P. Phase III study of pemetrexed in combination with cisplatin versus cisplatin alone in patients with malignant pleural mesothelioma. J Clin Oncol (2003) 21:2636–44. doi: 10.1200/JCO.2003.11.136 12860938

[7] Fennell DA Ewings S Ottensmeier C Califano R Hanna GG Hill K. Nivolumab versus placebo in patients with relapsed malignant mesothelioma (CONFIRM): A multicentre, double-blind, randomised, phase 3 trial. Lancet Oncol (2021) 22:1530–40. doi: 10.1016/S1470-2045(21)00471-X PMC8560642 34656227

[8] Baas P. Nivolumab plus ipilimumab should be the standard of care for first-line unresectable epithelioid mesothelioma. J Thorac Oncol (2022) 17:30–3. doi: 10.1016/j.jtho.2021.07.029 34930609

[9] Zalcman G Mazieres J Margery J Greillier L Audigier-Valette C Moro-Sibilot D. Bevacizumab for newly diagnosed pleural mesothelioma in the mesothelioma avastin cisplatin pemetrexed study (MAPS): A randomised, controlled, open-label, phase 3 trial. Lancet (2016) 387:1405–14. doi: 10.1016/S0140-6736(15)01238-6 26719230

[10] Désage AL Karpathiou G Peoc’h M Froudarakis ME. The immune microenvironment of malignant pleural mesothelioma: A literature review. Cancers (Basel) (2021) 13:1–31. doi: 10.3390/cancers13133205 PMC8269097 34206956

[11] Baas P Scherpereel A Nowak AK Fujimoto N Peters S Tsao AS. First-line nivolumab plus ipilimumab in unresectable malignant pleural mesothelioma (CheckMate 743): a multicentre, randomised, open-label, phase 3 trial. Lancet (2021) 397:375–86. doi: 10.1016/S0140-6736(20)32714-8 33485464

[12] Fennell DA Dulloo S. Chemotherapy with or without bevacizumab should be the standard of care for first-line unresectable epithelioid mesothelioma. J Thorac Oncol (2022) 17:34–7. doi: 10.1016/j.jtho.2021.08.004 34930610

[13] Chéné AL D’Almeida S Blondy T Tabiasco J Deshayes S Fonteneau JF. Pleural effusions from patients with mesothelioma induce recruitment of monocytes and their differentiation into M2 macrophages. J Thorac Oncol (2016) 11:1765–73. doi: 10.1016/j.jtho.2016.06.022 27418105

[14] Lievense LA Cornelissen R Bezemer K Kaijen-Lambers MEH Hegmans JPJJ Aerts JGJV. Pleural effusion of patients with malignant mesothelioma induces macrophage-mediated T cell suppression. J Thorac Oncol (2016) 11:1755–64. doi: 10.1016/j.jtho.2016.06.021 27418106

[15] Hamaidia M Gazon H Hoyos C Hoffmann GB Louis R Duysinx B. Inhibition of EZH2 methyltransferase decreases immunoediting of mesothelioma cells by autologous macrophages through a PD-1-dependent mechanism. JCI Insight (2019) 4:1–17. doi: 10.1172/jci.insight.128474 PMC6795292 31534051

[16] Gauttier V Pengam S Durand J Biteau K Mary C Morello A. Selective SIRPα blockade reverses tumor T cell exclusion and overcomes cancer immunotherapy resistance. J Clin Invest (2020) 130:6109–23. doi: 10.1172/JCI135528 PMC7598080 33074246

[17] Mola S Pinton G Erreni M Corazzari M De Andrea M Grolla AA. Inhibition of the histone methyltransferase EZH2 enhances protumor monocyte recruitment in human mesothelioma spheroids. Int J Mol Sci (2021) 22:1–25. doi: 10.3390/ijms22094391 PMC8122808 33922336

[18] Hoyos C Fontaine A Jacques JR Heinen V Louis R Duysinx B. HDAC inhibition with valproate improves direct cytotoxicity of monocytes against mesothelioma tumor cells. Cancers (Basel) (2022) 14:1–19. doi: 10.3390/cancers14092164 PMC9100202 35565292

[19] Reichman H Karo-atar D Munitz A. Emerging roles for eosinophils in the tumor microenvironment. Trends Cancer (2016) 2:664–75. doi: 10.1016/j.trecan.2016.10.002 28741505

[20] Varricchi G Galdiero MR Loffredo S Lucarini V Marone G Mattei F. Eosinophils: The unsung heroes in cancer? Oncoimmunology (2018) 7:1–14. doi: 10.1080/2162402X.2017.1393134 PMC5749653 29308325

[21] Scherpereel A Opitz I Berghmans T Psallidas I Glatzer M Rigau D. ERS/ESTS/EACTS/ESTRO guidelines for the management of malignant pleural mesothelioma. Eur Respir J (2020) 55:1–31. doi: 10.1183/13993003.00953-2019 32451346

[22] Byrne MJ Nowak AK. Modified RECIST criteria for assessment of response in malignant pleural mesothelioma. Ann Oncol (2004) 15:257–60. doi: 10.1093/annonc/mdh059 14760119

[23] Camp RL Dolled-Filhart M Rimm DL. X-Tile: A new bio-informatics tool for biomarker assessment and outcome-based cut-point optimization. Clin Cancer Res (2004) 10:7252–9. doi: 10.1158/1078-0432.CCR-04-0713 15534099

[24] Santoro A O’Brien ME Stahel RA Nackaerts K Baas P Karthaus M. Pemetrexed plus cisplatin or pemetrexed plus carboplatin for chemonaïve patients with malignant pleural mesothelioma: Results of the international expanded access program. J Thorac Oncol (2008) 3:756–63. doi: 10.1097/JTO.0b013e31817c73d6 18594322

[25] Van Meerbeeck JP Gaafar R Manegold C Van Klaveren RJ Van Marck EA Vincent M. Randomized phase III study of cisplatin with or without raltitrexed in patients with malignant pleural mesothelioma: An intergroup study of the European organisation for research and treatment of cancer lung cancer group and the national cancer institute. J Clin Oncol (2005) 23:6881–9. doi: 10.1200/JCO.20005.14.589 16192580

[26] Kovalszki A Weller PF. Eosiniophilia. Prim Care (2016) 43:607–17. doi: 10.1016/j.pop.2016.07.010.Eosinophilia PMC5293177 27866580

[27] Kovalszki A Weller PF. “Eosinophils and eosinophilia“, In: Rich RR Fleisher TA Shearer WT Schroeder HW Frew A Weyand CM, editors. Clinical Immunology Principles and Practices (Fifth Edition) (2019), p. 349–61. doi: 10.1016/B978-0-7020-6896-6.00024-7

[28] U.S. Food and Drug Administration. Clinical trial endpoints for the approval of cancer drugs and biologics: Guidance for industry (Rockville: U.S. Food and Drug Administration) (2018). pp. 1–16.

[29] Yamazoe M Ozasa H Kim YH. Effectiveness of nivolumab on sarcomatoid malignant pleural mesothelioma with eosinophilia and eosinophilic pleural effusion. J Thorac Oncol (2019) 14:e251–3. doi: 10.1016/j.jtho.2019.06.007 31668324

[30] Takeuchi E Takahashi N Morizumi S Tamiya H Matsuoka H Kuroda N. Interleukin-5-producing malignant pleural mesothelioma with eosinophilic pleural effusion. Thorac Cancer (2020) 11:3043–6. doi: 10.1111/1759-7714.13652 PMC7529565 32894005

[31] Yamazaki M Ohwada A Miyaji A Yamazaki H Nara T Hirai S. Pulmonary paragonimiasis with coincidental malignant mesothelioma. Intern Med (2008) 47:1027–31. doi: 10.2169/internalmedicine.47.0852 18520115

[32] Grisaru-Tal S Itan M Klion AD Munitz A. A new dawn for eosinophils in the tumour microenvironment. Nat Rev Cancer (2020) 20:594–607. doi: 10.1038/s41568-020-0283-9 32678342

[33] Simon SCS Utikal J Umansky V. Opposing roles of eosinophils in cancer. Cancer Immunol Immunother (2019) 68:823–33. doi: 10.1007/s00262-018-2255-4 PMC11028063 30302498

[34] Wu HX Zhuo KQ Cheng DY. Peripheral blood eosinophil as a biomarker in outcomes of acute exacerbation of chronic obstructive pulmonary disease. Int J COPD (2019) 14:3003–15. doi: 10.2147/COPD.S226783 PMC6935282 31920297

[35] Zhang Y Liang LR Zhang S Lu Y Chen YY Shi HZ. Blood eosinophilia and its stability in hospitalized COPD exacerbations are associated with lower risk of all-cause mortality. Int J COPD (2020) 15:1123–34. doi: 10.2147/COPD.S245056 PMC7245431 32547000

[36] Reichman H Itan M Rozenberg P Yarmolovski T Brazowski E Varol C. Activated eosinophils exert antitumorigenic activities in colorectal cancer. Cancer Immunol Res (2019) 7:388–400. doi: 10.1158/2326-6066.CIR-18-0494 30665890

[37] Onesti CE Josse C Boulet D Thiry J Beaumecker B Bours V. Blood eosinophilic relative count is prognostic for breast cancer and associated with the presence of tumor at diagnosis and at time of relapse. Oncoimmunology (2020) 9:1–11. doi: 10.1080/2162402X.2020.1761176 PMC7458605 32923121

[38] Günduz S Göksu SS Arslan D Tatli AM Uysal M Gündüz UR. Factors affecting disease-free survival in patients with human epidermal growth factor receptor 2-positive breast cancer who receive adjuvant trastuzumab. Mol Clin Oncol (2015) 3:1109–12. doi: 10.3892/mco.2015.610 PMC4534854 26623060

[39] Steel JL Kim KH Dew MA Unruh ML Antoni MH Olek MC. Cancer-related symptom clusters, eosinophils, and survival in hepatobiliary cancer: An exploratory study. J Pain Symptom Manag (2010) 39:859–71. doi: 10.1016/j.jpainsymman.2009.09.019 PMC3127169 20471546

[40] Davis BP Rothenberg ME. Eosinophils and cancer. Cancer Immunol Res (2014) 2:1–9. doi: 10.1158/2326-6066.CIR-13-0196 24778159

[41] Simon SCS Hu X Panten J Grees M Renders S Thomas D. Eosinophil accumulation predicts response to melanoma treatment with immune checkpoint inhibitors. Oncoimmunology (2020) 9:1–12. doi: 10.1080/2162402X.2020.1727116 PMC7028332 32117594

[42] Moreira A Leisgang W Schuler G Heinzerling L. Eosinophilic count as a biomarker for prognosis of melanoma patients and its importance in the response to immunotherapy. Immunotherapy (2017) 9:115–21. doi: 10.2217/imt-2016-0138 28128709

[43] Wei Y Zhang X Wang G Zhou Y Luo M Wang S. The impacts of pretreatment circulating eosinophils and basophils on prognosis of stage I–III colorectal cancer. Asia Pac J Clin Oncol (2018) 14:e243–51. doi: 10.1111/ajco.12871 29532611

[44] Tancrède-Bohin E Ionescu MA de la Salmonière P Dupuy A Rivet J Rybojad M. Prognostic value of blood eosinophilia in primary cutaneous T-cell lymphomas. Arch Dermatol (2004) 140:1057–61. doi: 10.1001/archderm.140.9.1057 15381544

[45] Utsunomiya A Ishida T Inagaki A Ishii T Yano H Komatsu H. Clinical significance of a blood eosinophilia in adult T-cell leukemia/lymphoma: A blood eosinophilia is a significant unfavorable prognostic factor. Leuk Res (2007) 31:915–20. doi: 10.1016/j.leukres.2006.10.017 17123603

[46] Bishara S Griffin M Cargill A Bali A Gore ME Kaye SB. Pre-treatment white blood cell subtypes as prognostic indicators in ovarian cancer. Eur J Obstet Gynecol Reprod Biol (2008) 138:71–5. doi: 10.1016/j.ejogrb.2007.05.012 17644243

[47] Okauchi S Shiozawa T Miyazaki K Nishino K Sasatani Y Ohara G. Association between peripheral eosinophils and clinical outcomes in patients with non-small cell lung cancer treated with immune checkpoint inhibitors. Polish Arch Intern Med (2021) 131:152–60. doi: 10.20452/pamw.15776 33491942

[48] Alves A Dias M Campainha S Barroso A. Peripheral blood eosinophilia may be a prognostic biomarker in non-small cell lung cancer patients treated with immunotherapy. J Thorac Dis (2021) 13:2716–27. doi: 10.21037/jtd-20-3525 PMC8182546 34164164

[49] Januskevicius A Jurkeviciute E Janulaityte I Kalinauskaite-Zukauske V Miliauskas S Malakauskas K. Blood eosinophils subtypes and their survivability in asthma patients. Cells (2020) 9:1–17. doi: 10.3390/cells9051248 PMC7291159 32443594

[50] Mesnil C Raulier S Paulissen G Xiao X Birrell MA Pirottin D. Lung-resident eosinophils represent a distinct regulatory eosinophil subset. J Clin Invest (2016) 126:3279–95. doi: 10.1172/JCI85664 PMC5004964 27548519

[51] Percopo CM Brenner TA Ma M Kraemer LS Hakeem RMA Lee JJ. SiglecF + Gr1 hi eosinophils are a distinct subpopulation within the lungs of allergen-challenged mice. J Leukoc Biol (2017) 101:321–8. doi: 10.1189/jlb.3a0416-166r PMC5166438 27531929

[52] Gutierrez-Sainz L Cruz P Martinez-Recio S Higuera O Esteban-Rodriguez MI Arias-Lotto F. Malignant pleural mesothelioma: clinical experience and prognostic value of derived neutrophil-to-lymphocyte ratio and PD-L1 expression. Clin Transl Oncol (2021) 23:2030–5. doi: 10.1007/s12094-021-02605-w 33837910

[53] Urso L Silic-Benussi M Boscolo A Lorenzi M Bonanno L Lunardi F. Detection of circulating immunosuppressive cytokines in malignant pleural mesothelioma patients for prognostic stratification. Cytokine (2021) 146:155622. doi: 10.1016/j.cyto.2021.155622 34153874

[54] De Fonseka D Arnold DT Morley AJ Brett M Bhatt N Edey A. Lymphocyte predominance in blood, pleural fluid, and tumour stroma; a prognostic marker in pleural mesothelioma. BMC Pulm Med (2022) 22:1–6. doi: 10.1186/s12890-022-01968-2 35501755 PMC9063088

[55] Cimen F Agackiran Y Düzgün S Aloglu M Senturk A Atikcan S. Factors affecting the life expectancy in malignant pleural mesothelioma: Our 10 years of studies and experience. Med (Baltimore) (2022) 101:e30711. doi: 10.1097/md.0000000000030711 PMC9524951 36181042

[56] Okita R Okada M Inokawa H Murakami T Ikeda E. Prognostic values of preoperative c-reactive protein, albumin, and neutrophil ratios in patients with malignant pleural mesothelioma who underwent extrapleural pneumonectomy. Surg Oncol (2022) 43:101813. doi: 10.1016/j.suronc.2022.101813 35816852

[57] Fournel L Charrier T Huriet M Iaffaldano A Lupo A Damotte D. Prognostic impact of inflammation in malignant pleural mesothelioma: A large-scale analysis of consecutive patients. Lung Cancer (2022) 166:221–7. doi: 10.1016/j.lungcan.2022.03.014 35334416

[58] Hazarika M White RM Booth BP Wang Y Ham DYL Liang CY. Pemetrexed in malignant pleural mesothelioma. Clin Cancer Res (2005) 11:982–92. doi: 10.1158/1078-0432.982.11.3 15709163

[59] Sakurada T Kakiuchi S Tajima S Horinouchi Y Konaka K Okada N. Pemetrexed-induced rash may be prevented by supplementary corticosteroids. Biol Pharm Bull (2015) 38:1752–6. doi: 10.1248/bpb.b15-00435 26521826

[60] Sakurada T Nokihara H Koga T Zamami Y Goda M Yagi K. Prevention of pemetrexed-induced rash using low-dose corticosteroids: A phase II study. Oncologist (2022) 27:e554–60. doi: 10.1093/oncolo/oyab077 PMC9255977 35325241

[61] Cook AM McDonnell AM Lake RA Nowak AK. Dexamethasone co-medication in cancer patients undergoing chemotherapy causes substantial immunomodulatory effects with implications for chemo-immunotherapy strategies. Oncoimmunology (2016) 5:1–11. doi: 10.1080/2162402X.2015.1066062 PMC4839331 27141331

[62] Cusack RP Whetstone CE Xie Y Ranjbar M Gauvreau GM. Regulation of eosinophilia in asthma–new therapeutic approaches for asthma treatment. Cells (2021) 10:1–23. doi: 10.3390/cells10040817 PMC8067385 33917396

