# Supplementary figures and images for: Excess of blood eosinophils prior to therapy correlates with worse prognosis in mesothelioma
Source: Front Immunol. 2023 Mar 21;14:1148798. doi: 10.3389/fimmu.2023.1148798 (PMC10070849; doi:10.3389/fimmu.2023.1148798)

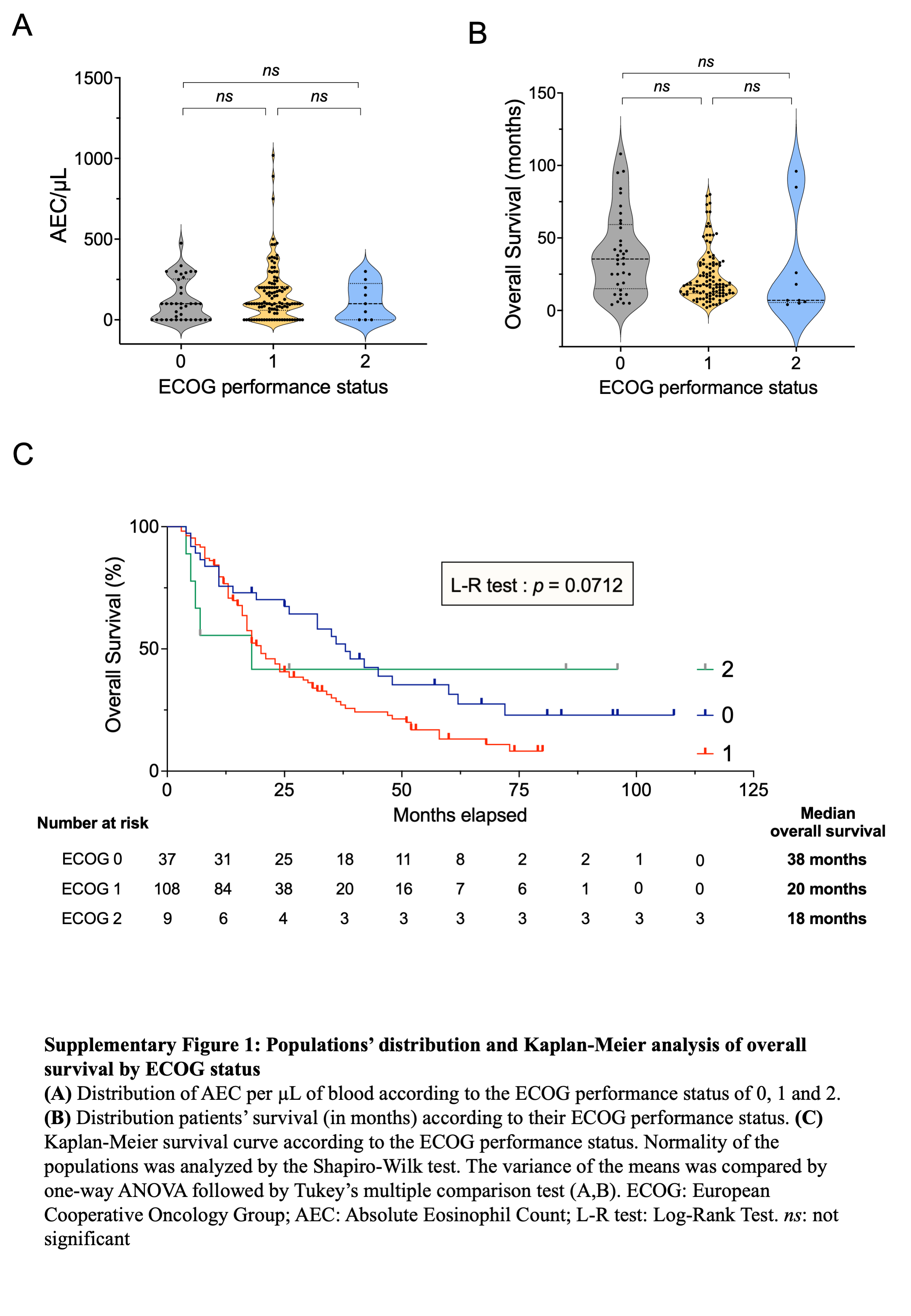

Supplement: Supplementary file 1 [file Image_1.tiff]

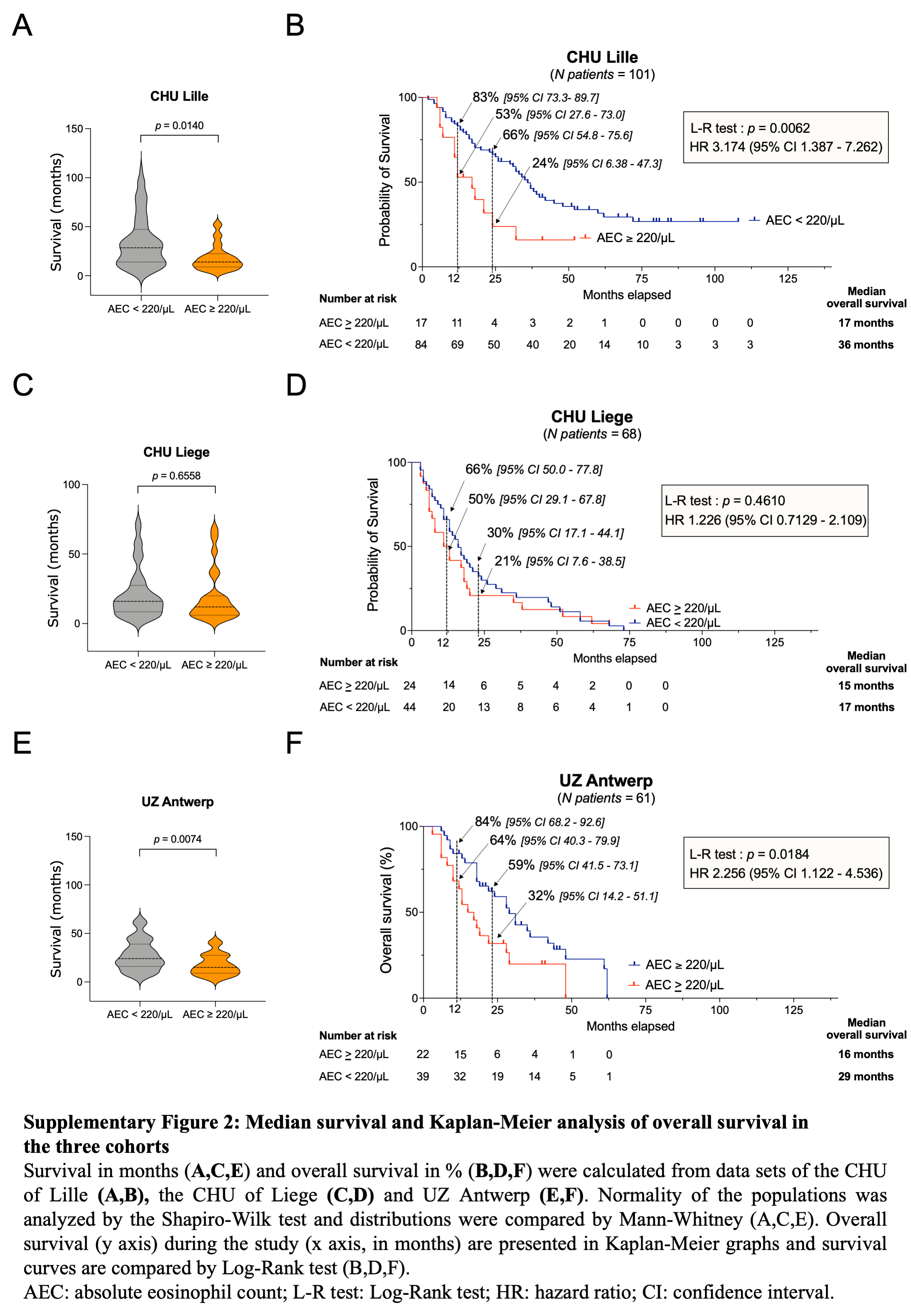

Supplement: Supplementary file 2 [file Image_2.tiff]

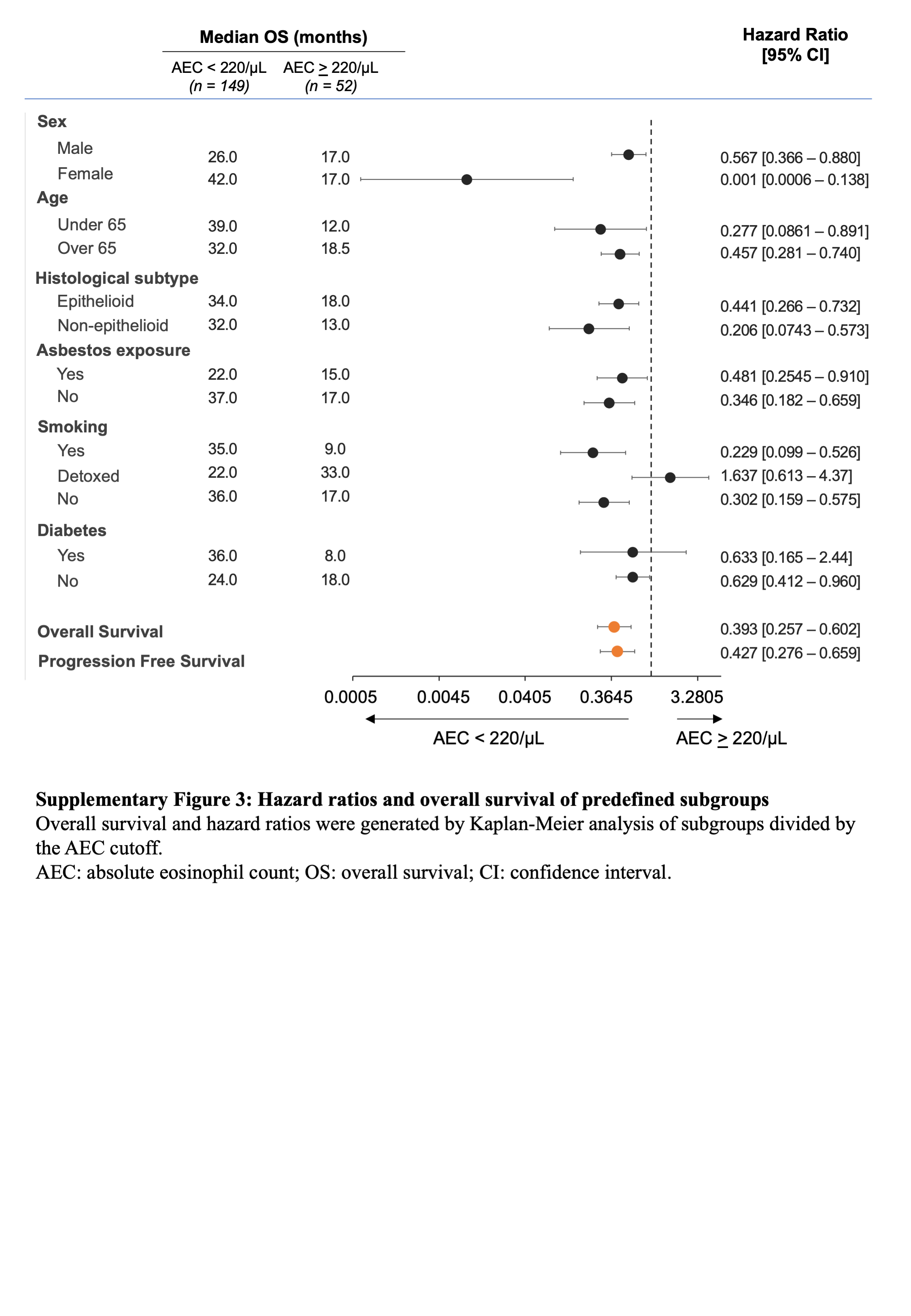

Supplement: Supplementary file 3 [file Image_3.tiff]

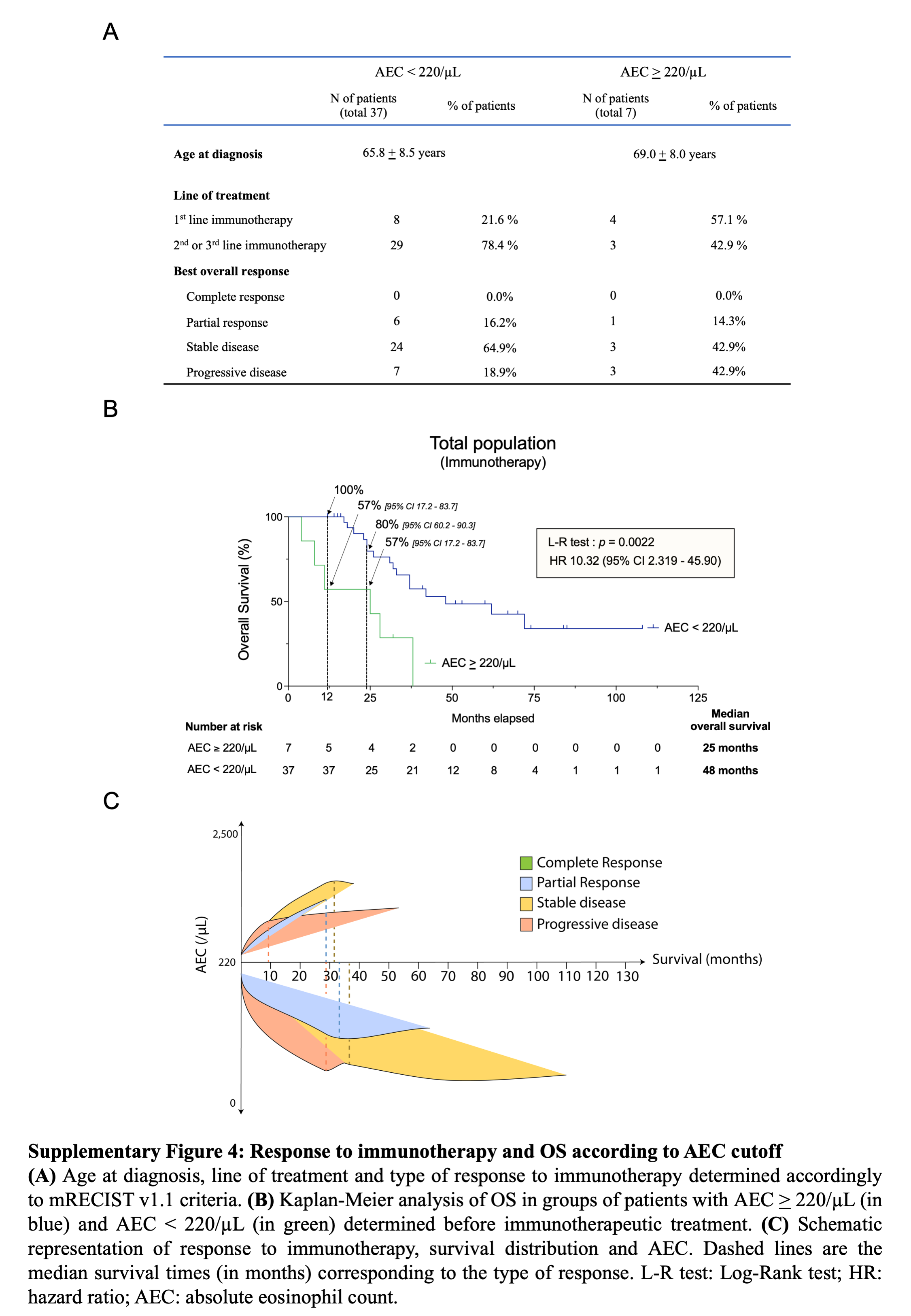

Supplement: Supplementary file 4 [file Image_4.tiff]

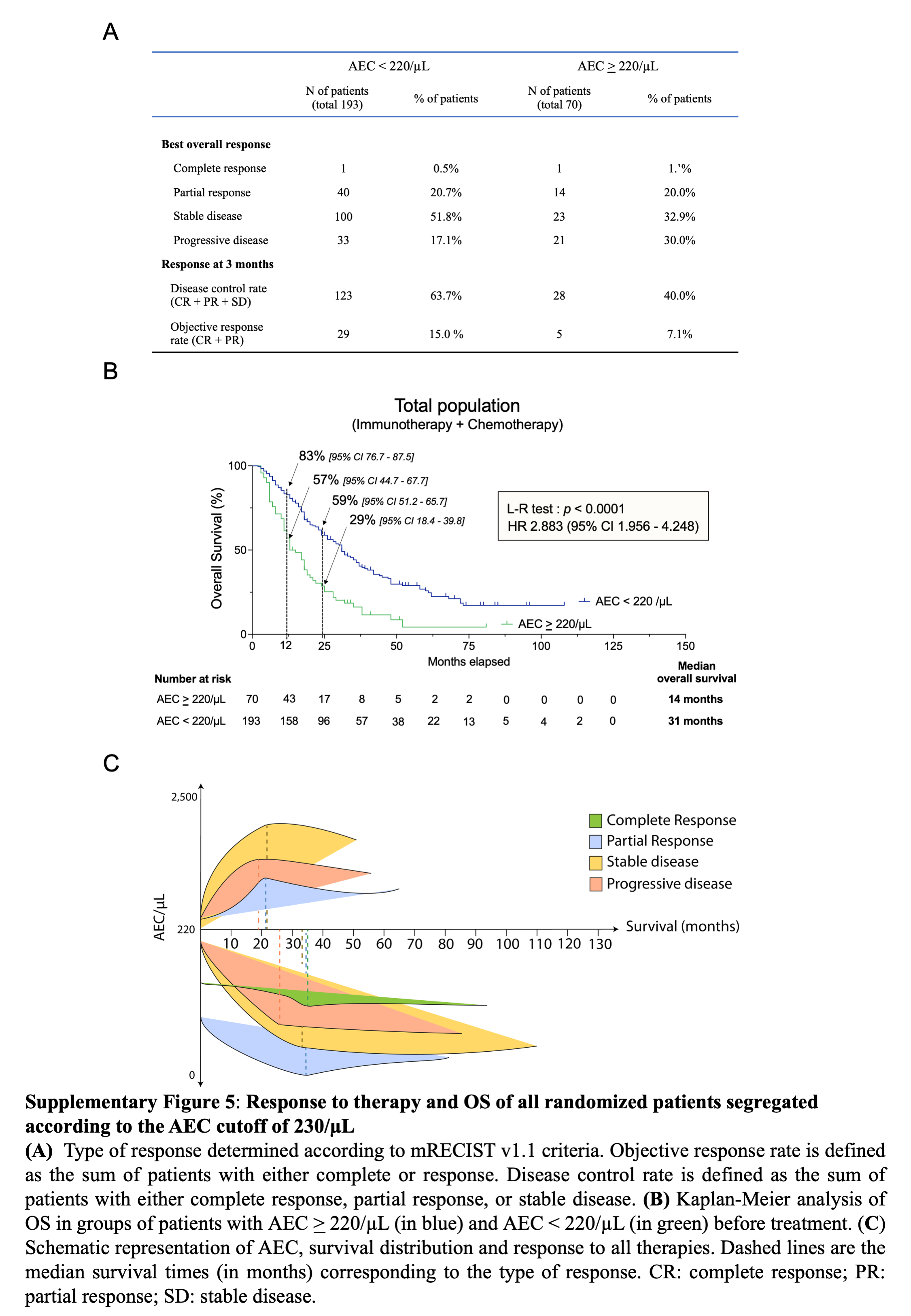

Supplement: Supplementary file 5 [file Image_5.tiff]
