# Supplementary material for: Excess of blood eosinophils prior to therapy correlates with worse prognosis in mesothelioma
Source: Front Immunol. 2023 Mar 21;14:1148798. doi: 10.3389/fimmu.2023.1148798 (PMC10070849; doi:10.3389/fimmu.2023.1148798)
Supplement: Supplementary file 6 [file Table_1.pdf]

Supplementary Table 1: Comparison of patients' characteristics in the three cohorts (CHU Lille, CHU Liege, UZ Antwerp) included in the study

	**CHU Lille** *(101 patients)*		**CHU Liege** *(68 patients)*		**UZ Antwerp** *(61 patients)*	
	AEC < 220/µL	AEC ≥ 220/µL	AEC < 220/µL	AEC ≥ 220/µL	AEC < 220/µL	AEC ≥ 220/µL
	**83** (82.2%)	**18** (17.8%)	**46** (67.65%)	**22** (32.35%)	**40** (65.6%)	**21** (34.4%)
**Sex, n**						
Male	56	16	41	18	28	20
Female	27	2	5	4	12	2
**Histological subtype, n**						
Epithelioid	71	14	34	18	39	16
Non-epithelioid	10	4	8	4	1	5
Sarcomatoid	5	2	1	0	0	3
Biphasic	4	2	5	3	1	2
Desmoplasmic	1	0	2	1	0	0
Unknown	2	0	4	0	0	0
**ECOG performance status, n**						
0	23	6	5	2	N/A	N/A
1	47	10	37	16	N/A	N/A
2	5	2	2	0	N/A	N/A
Unknown	0	0	1	4	41	20

n = number of patients
